# Facebook-Based Social Marketing to Reduce Smoking in Australia’s First Nations Communities: An Analysis of Reach, Shares, and Likes

**DOI:** 10.2196/16927

**Published:** 2020-12-10

**Authors:** Marita Hefler, Vicki Kerrigan, Anne Grunseit, Becky Freeman, James Kite, David P Thomas

**Affiliations:** 1 Menzies School of Health Research Charles Darwin University Casuarina Australia; 2 Prevention Research Collaboration Sydney School of Public Health The University of Sydney Sydney Australia

**Keywords:** social media, tobacco, Australia, indigenous peoples, smoking, health promotion

## Abstract

**Background:**

Facebook is widely used by Australia’s First Nations people and has significant potential to promote health. However, evidence-based guidelines for its use in health promotion are lacking. Smoking prevalence among Australia’s First Nations people is nearly 3 times higher than other Australians. Locally designed programs in Aboriginal Community Controlled Health Services (ACCHOs) to reduce smoking often use Facebook.

**Objective:**

This study reports on an analysis of the reach and engagement of Facebook posts with smoking prevention and cessation messages posted by ACCHOs in the Northern Territory, Australia.

**Methods:**

Each service posted tobacco control content at least weekly for approximately 6 months. Posts were coded for the following variables: service posted, tailored First Nations Australian content, local or nonlocally produced content, video or nonvideo, communication technique, and emotional appeal. The overall reach, shares, and reactions were calculated.

**Results:**

Compared with posts developed by the health services, posts with content created by other sources had greater reach (adjusted incident rate ratio [IRR] 1.92, 95% CI 1.03-3.59). Similarly, reactions to posts (IRR 1.89, 95% CI 1.40-2.56) and shared posts (IRR 2.17, 95% CI 1.31-3.61) with content created by other sources also had more reactions, after controlling for reach, as did posts with local First Nations content compared with posts with no First Nations content (IRR 1.71, 95% CI 1.21-2.34).

**Conclusions:**

Facebook posts with nonlocally produced content can be an important component of a social media campaign run by local health organizations. With the exception of nonlocally produced content, we did not find a definitive set of characteristics that were clearly associated with reach, shares, and reactions. Beyond reach, shares, and likes, further research is needed to understand the extent that social media content can influence health behavior.

## Introduction

### Social Media and Health

Social media are an essential component of public health, as a tool for health communication, an avenue for consumers seeking information, and an environment that shapes health, analogous to the influence of the built environment [1]. Although social media have fundamentally altered health promotion [2], considerable uncertainty remains regarding how health services can use them effectively [3,4]. The rapid change in the social media landscape challenges the capacity of health promoters to develop a robust evidence base for what works; as a result, there are no clear guidelines for population-based social media strategies to promote health. Numerous reviews have concluded that social media have the potential to positively affect health but have found either mixed [5,6] or no definitive evidence [7-11] of their impact on health behavior, even as research in the field has significantly increased in recent years.

Consumer benefits of using social media for health information include social and emotional support and connectedness from peer-to-peer interactions [12-14]. Similarly, social media are an opportunity for health services to engage in building social capital within their communities [15] and a potentially cost-effective way to achieve significant reach for social marketing and health promotion [3]. Social media may also be effective for engaging specific groups who experience disadvantage and, therefore, contribute to reducing health inequity, although more research is needed [10].

Social marketing and mass media are an important and cost-effective strategy for reducing smoking [16-19]. Social media are an increasingly integral component of mass media strategies, and Australian research suggests that social media alone are more cost-effective than television advertising for tobacco control social marketing [20]. Social media also complement strategies such as smoking cessation services [21]. Facebook can also be effective for connecting with otherwise hard-to-reach smokers [22], including with people who are not social media users when people show content that appears on their Facebook feed to people who do not have an account [15].

Worldwide, Facebook is the largest social media platform, with nearly 2.8 billion active users [23]. In Australia, it is the most widely and frequently used platform, with 15 million active users (equivalent to 60% of the population) and 50% of the population using it daily [24]. Facebook is a comparatively inexpensive way for health services to communicate with the public [25], allowing for a spectrum of engagement from low to high [26]. Unlike many other platforms, Facebook is used by all ages, including older adults, for whom it can have both social and health benefits [13,14,27]. Studies examining the characteristics of posts that generate engagement have found diverse results; some have found videos to be effective [28], others have found photos to be effective [29], and some have identified that paid boosts and page promotions stimulate engagement [26,30] and that the time of day or day of week and organic versus paid posts are also factors that determine both reach and engagement [30]. The Facebook algorithm appears to increasingly prioritize posts from personal contacts rather than unpaid posts from organizational or business pages [31]. In this context, health services need to understand how they can use Facebook to reach key target groups and track both reach and engagement.

### Social Media and Australia’s First Nations People

Although data are limited, social media—and specifically Facebook—use appears to be higher among Australia’s First Nations people than other Australians [32-34]. Facebook has been found to be a supportive environment which is used by Australia’s First Nations people to share health information [35,36]. The high use of social media by Australia’s First Nations peoples is not limited to urban areas; particularly in remote communities, Australia’s First Nations people are likely to be *mobile only* users in relation to communication, with social media access often occurring through shared devices [37].

Unsurprisingly, the lack of clear evidence about how to use social media effectively to improve health is reflected in reviews about how social media can be used to improve the health of Australia’s First Nations people [36,38]. However, research is promising that social media can help with promoting healthy behaviors [39]. In Australia, the use of social media by First Nations people has some specific characteristics, including engagement between youth and older adults, which promotes intergenerational connection [33]. Social media is therefore an appropriate channel for communicating messages to Australia’s First Nations people as part of social marketing and mass media campaigns.

### Australia’s First Nations People and Smoking

Nationally, smoking prevalence among Australia’s First Nations people is nearly 3 times higher than that among non-Indigenous Australians [40]. To address this, the national Tackling Indigenous Smoking Program is funded by the Australian government till June 30, 2022. It is implemented through 37 Aboriginal Community Controlled Health Services (ACCHOs) to deliver locally designed programs to reduce smoking in their communities [41] and includes the use of mass media, both legacy (television, radio, and newspaper) and social media. These local health services therefore play an essential role in disseminating information to localize and make relevant key tobacco control messages and generate awareness of cessation support for smokers.

### Study Objective

This study reports on an analysis of Facebook post reach and engagement with smoking prevention and cessation messages posted by ACCHOs in the Northern Territory, Australia.

A note regarding terminology: the original inhabitants of what is now known as Australia have diverse cultures, languages, and kinship structures. Throughout this paper, the term Australia’s First Nations is used, unless referring specifically to service or program names that use Indigenous or Aboriginal in the title.

## Methods

### Setting

In 2016, the Northern Territory had an estimated population of 245,048 people [42]. The Northern Territory has both the highest proportion of First Nations people (23.8%) [42] and the highest smoking prevalence compared with any other Australian jurisdiction. Among people aged 18 years or older, the daily smoking prevalence in the Northern Territory in 2017 to 2018 was 19.6%, compared with 13.8% of all Australians [43]. In 2014, the daily smoking prevalence among First Nations people in the Northern Territory was 45% [44].

This study was undertaken with 3 ACCHOs in the Northern Territory, located in a mix of urban and remote locations. It was designed to meet an identified need for services to have a better, context-specific understanding of what content works well with their communities. Implementation was participatory, flexible, and pragmatic, based on real-world circumstances for, and decision making by, each service (rather than imposing a set approach to selecting and posting content). All the services received funding from the Tackling Indigenous Smoking Program. The approach and factors explored in this study were based on guidance from the services that were partners in this project and feedback from other services that receive Tackling Indigenous Smoking Program funding. One participating service (hereafter referred to as service 3) had an organizational Facebook page before the commencement of the study; the other 2 started their Facebook pages during the study. Data were collected from the time of the first tobacco-related post during the study period. Each service posted a mix of tobacco control content, other health information, and general information during the study period. Data collected and analyzed for this study only included tobacco-related posts. One health service paid to boost the reach of 2 posts. With only 2 posts, it was not possible to assess the impact of these posts on reach.

One health service that started its Facebook page during the study had an in-house specialist communications manager who was responsible for coordinating and managing posts for this study, as part of managing social media communications for the service. The other 2 services received support and mentoring from VK (project manager with the research institute) throughout the study period, including technical assistance to develop content, support for crafting messages, and risk management for negative public comments. This technical support was part of the overall project design, as requested by the partner services. VK has 2 decades of experience as a journalist, including administration of local Facebook accounts for a national media outlet. The support provided by VK was part of capacity building and reciprocity, which was embedded in the project approach, in line with Australian national guidelines for ethical conduct in Aboriginal and Torres Strait Islander health research [45]. The project was approved by the Northern Territory Department of Health and Menzies School of Health Research Human Research Ethics Committee and the Central Australian Human Research Ethics Committee, although ethics approval was not required for this component.

### Data Collection

Facebook analytic data (available to page administrators) were collected for the duration of each health service’s participation in the project. Data were collected 7 to 14 days after each post. The difference in time between posting and data collection is unlikely to have affected the overall reach, as most reach and interactions with Facebook posts are known to be achieved within 24 hours of posting [46]. The study period and timeframe were different for each service to take into account operational priorities, staff capacity, and holiday close down periods.

### Outcome Variables

We collected the following outcome measures for each post:

- Overall reach: the total number of unique users exposed to the original post and shares, as defined by Facebook. Facebook insights do not provide a breakdown of exposure to the original post and shares.
- Total shares: includes shares of the original post and on-shares (shares of the shared post). This category also includes shares of posts that the health service posted on its own page and then shared to the page of another organization.
- Reactions to original post: all reactions (like, love, laugh, angry, sad, and wow) and comments on a post by the health service. Does not include shares of the post or reactions to on-shares.
- Reactions to shared posts: all reactions (like, love, laugh, angry, sad, and wow) and comments to posts from the health service that were shared by others.

Reactions to original posts and shared posts were analyzed separately to provide insight into differences in the magnitude of reactions to original posts compared with shared posts. Furthermore, it was not always possible to determine what was included in shared posts—for example, comments by the health services on the original post may not have been included or people may have added their own comments when sharing posts that were not visible to the research team.

### Independent Variables

We adapted previous coding frames [28,47] for post content to take into account the smaller volume of posts analyzed in this study and ensure that codes were locally relevant. Coding criteria were determined by agreement between 3 authors (VK, MH, and DT), with potential discrepancies resolved at this stage. Codes were then assigned by 1 author (VK; Textbox 1), except for *emotional appeal* (see definition in Textbox 1).

**Coding frame for post content (item and definition).**

- Who posted: Health service 1, 2, or 3
- First Nations (Australia) tailoring:
  Non–First Nations: General tobacco control messages, whether Australian or international, not featuring any First Nations people, words or images
  First Nations Australia—general: Australian content, tailored to First Nations people, but not specific to the health service location. Includes content from locations in the Northern Territory, but outside the catchment area or footprint of the health service, and content developed by other services in the project
  First Nations Australia—local: Content which has features that are localized to health service area. Includes First Nations Australia general content with local branding features such as local health service logo or translated into language
- Content origin:
  Original: Content developed by the health service which posted the content
  Other sources: Content created by a source other than the health service, even if localized to the health service (eg, Australian Government Department of Health “Don’t make smokes your story” campaign images translated into the local language and/or with a logo of a local health service added)
- Post type: Includes or does not include video
- Communication techniques:
  Other orgs tagged—no or yes
  Hashtags used—yes or no
- Emotional appeal: As this is influenced by cultural background, posts from each health service were coded by 2 community members living in the catchment area of each service. Coders were requested to code each post as negative, positive, or neutral. Coding was based on their perceptions of the post; the intended emotional appeal of the content was not known by the researchers or coders. If coders perceived the post as having no emotional elements or both positive and negative elements, they were asked to code it as neutral. Posts that were coded differently by each community member were combined with those that were coded as neutral by both. As no posts were coded as negative by the coders, 2 final categories were used for analysis: (1) positive and (2) neutral or disagreement (contested) between coders

First, we calculated the number of posts, shares, reactions, and median reach per post over the independent variables. Second, we conducted a series of (separate) bivariate negative binomial regressions (the data were overdispersed) for each outcome (reach, shares, reactions to original post, and reaction to shared posts) over each of the 6 explanatory variables, and then multivariate regressions including all of the explanatory variables. The reference category for health service was health service 1. For First Nations tailoring, it was non–First Nations content; for content origin, it was original content; for post type, it was nonvideo; and for whether other organizations were tagged or hashtags were used, the reference categories were not tagged and no hashtags, respectively, and neutral or mixed was the reference category for emotional appeal.

Estimated coefficients for multicategory variables (health service and First Nations tailoring) were tested jointly postestimation for statistical significance of the variable using Wald tests. The analyses were initially conducted without an offset and then controlling for users’ exposure to the post by including reach as an offset variable to estimate engagement while accounting for the number of people to whom each post was delivered [48]. For the unadjusted analyses, the outcome (incident rate ratio [IRR]) indicates the multiplicative change in outcome compared with the reference category for that explanatory variable. For the adjusted analyses, the relationship between explanatory variables and engagement becomes the change in the outcome per person. The threshold for statistical significance was set at *P*=.05.

## Results

Service 1 participated for 23 weeks from August 2017, service 2 for 19 weeks from July 2017 to March 2018, and service 3 for 19 weeks from May to December 2017. We coded 92 posts from the study period (Table 1).

**Table 1:** Overall reach of each type of post (both of the original post and when shared).

Variable		Posts, n	Overall reach						
			Reach per post, median	IRR^a^	95% CI	*P* value^b^	Adjusted IRR^c^	95% CI	*P* value^b^
Total		92	248	N/A^d^	N/A	N/A	N/A	N/A	N/A
**Health service**						<.001			<.001
	1	33	177	1	N/A		1	N/A	
	2	25	114	0.94	0.56-1.59		1.11	0.61-2.04	
	3	34	1432	5.20	3.22-8.39		6.18	3.33-11.47	
**First Nations (Australia) content**						.11			.20
	No	20	448	1	N/A		1	N/A	
	Yes, not local	14	189	0.40	0.17-0.91		0.94	0.46-1.96	
	Yes, local	58	248	0.69	0.37-1.28		0.61	0.33-1.11	
**Content source**						.008			.04
	Original content	67	192	1	N/A		1	N/A	
	Other sources	25	1014	2.10	1.21-3.60		1.92	1.03-3.59	
**Video**						.02			.72
	No	70	244	1	N/A		1	N/A	
	Yes	22	762	1.95	1.10-3.46		0.90	0.49-1.64	
**Other organizations tagged**						.25			.34
	No	83	247	1	N/A		1	N/A	
	Yes	9	488	1.63	0.71-3.78		1.48	0.66-3.33	
**Hashtagged**						.33			.39
	No	14	312	1	N/A		1	N/A	
	Yes	78	248	1.42	0.71-2.84		1.37	0.67-2.80	
**Tone of content**						.19			.02
	Neutral or disagreement between coders^e^	48	244	1	N/A		1	N/A	
	Positive	44	294	1.39	0.85-2.29		0.51	0.29-0.90	

^a^IRR: incident rate ratio.

^b^*P* value calculated for whole variable using chi-square test.

^c^Adjusted IRR for all other variables describing types of posts using multiple negative binomial regression.

^d^N/A: not applicable.

^e^Includes posts where all coded as neutral content and posts where coders could not agree on them being positive, neutral, or negative. No posts were coded as negative in tone or content by all coders.

Most posts (58/92, 63%) featured local First Nations content (rather than either no Australian First Nations content or content that was not local) and original content created by the health service (67/92, 73% posts; rather than content from other sources). There were no posts that featured content that was perceived as negative by all coders, such as intended negative emotional arousal featured in fear appeal TV campaigns; there was disagreement between coders on 37 posts, and the remainder were coded by all coders as either positive (44 posts) or neutral (11 posts). As noted in Textbox 1, neutral and posts with coder disagreement were treated as 1 category. Only 22 of 92 posts (24%) included videos, and although most (78/92, 85%) included hashtags, fewer than 10% tagged other organizations. One service paid to boost reach for 2 posts; all other posts (90/92, 97%) were organic (nonpaid page posts).

The median overall reach of each post was 248 unique users. Posts reached more people if they were from health service 3, which had an established Facebook page preproject, if the featured content was from other sources (rather than original content created by the health service) or if the posts contained videos. Although there was no association with reach in the bivariate analysis, once adjusted for other post features, having positive content was associated with less reach (compared with neutral or contested content). In relation to the service that boosted the reach of 2 posts, we conducted a sensitivity analysis of our results by removing the 2 paid posts; the same variables had significant associations in all tables.

The 92 posts were shared 352 times (median 1 share per post; IQR 0-4; range 0-45; Table 2). Posts from health services 2 and 3 were shared more than those from health service 1. Other characteristics were not associated with the sharing of posts (Multimedia Appendix 1).

**Table 2:** Total shares of each type of post, with and without controlling for reach.

Variable			Shares, n		Shares, Median		IRR^a^		95% CI		*P* value^b^		IRR (controlling for reach)^c^		95% CI		*P* value^b^
Total			352		1		N/A^d^		N/A		N/A		N/A		N/A		N/A
**Health service**											<.001						.005
	1	41		0		1		N/A				1		N/A			
	2	61		1		1.96		0.85-4.56				2.90		1.43-5.88			
	3	250		6		5.92		2.76-12.67				2.77		1.47-5.21			
**First Nations (Australia) content**											.22						.45
	No	127		2		1		N/A				1		N/A			
	Yes, not local	48		0		0.54		0.17-1.74				0.80		0.33-1.94			
	Yes, local	177		1		0.48		0.20-1.14				0.66		0.34-1.27			
**Content source**											.37						.95
	Original content	229		1		1		N/A				1		N/A			
	Other sources	123		4		1.44		0.65-3.20				1.02		0.56-1.88			
**Video**											.20						.23
	No	229		1		1		N/A				1		N/A			
	Yes	123		4		1.71		0.75-3.90				1.47		0.78-2.75			
**Other organizations tagged**											.82						.87
	No	313		1		1		N/A				1		N/A			
	Yes	39		4		1.15		0.34-3.83				1.08		0.44-2.65			
**Hashtagged**											.26						.58
	No	32		1		1		N/A				1		N/A			
	Yes	320		1		1.79		0.65-4.96				1.25		0.56-2.78			
**Tone of content**											.12						.37
	Neutral or disagreement between coders	135		1		1		N/A				1		N/A			
	Positive	217		1		1.75		0.86-3.56				1.29		0.74-2.24			

^a^IRR: incident rate ratio.

^b^*P* values calculated for whole variables using chi-square test. We controlled for reach by offsetting the negative binomial regression model by the reach of the original posts.

^c^We controlled for reach by offsetting the negative binomial regression model by the reach of the original posts.

^d^N/A: not applicable.

There were 1099 reactions to the original posts (median 7 reactions per post; IQR 3-13; range 0-94; Table 3). In the bivariate analyses, posts from health service 3 (compared with health service 1) with content from other sources (rather than original content created by the health service) featuring video and with other organizations tagged attracted more reactions (likes or comments). After controlling for the reach of the posts, however, the differences between health services became insignificant, and posts with local Australian First Nations content attracted more reactions per person reached (compared with posts with no Australian First Nations content).

After adjusting for all variables, posts with content from other sources (compared with posts with original content) attracted significantly more reactions (IRR 1.57, 95% CI 1.08-2.27; *P*=.02; Multimedia Appendix 2).

There were 990 reactions to the shared posts (median zero reactions per shared post; IQR 0-10; range 0-97; Table 4).

**Table 3:** Reactions to each type of original post, before and after controlling for reach.

Variable		Reactions, n	Reactions, Median	IRR^a^	95% CI	*P* value^b^	IRR (controlling for reach)^c^	95% CI	*P* value^b^
Total		1099	7	N/A^d^	N/A	N/A	N/A	N/A	N/A
**Health service**						<.001			.25
	1	334	4	1	N/A		1	N/A	
	2	162	5	0.64	0.38-1.08		1.30	0.88-1.94	
	3	603	10	1.75	1.09-2.81		0.95	0.67-1.35	
**First Nations (Australia) content**						.03			.003
	No	182	9	1	N/A		1	N/A	
	Yes, not local	92	5	0.72	0.35-1.48		0.96	0.59-1.58	
	Yes, local	825	6	1.56	0.93-2.64		1.71	1.19-2.46	
**Content source**						<.001			<.001
	Original content	517	4	1	N/A		1	N/A	
	Other sources	582	19	3.02	1.98-4.60		1.89	1.40-2.56	
**Video**						.002			*.*002
	No	661	6	1	N/A		1	N/A	
	Yes	438	11	2.11	1.31-3.40		1.68	1.21-2.34	
**Other organizations tagged**						.03			.02
	No	890	6	1	N/A		1	N/A	
	Yes	209	19	2.17	1.08-4.34		1.74	1.09-2.78	
**Hashtagged**						.83			.31
	No	158	7	1	N/A		1	N/A	
	Yes	941	8	1.07	0.59-1.95		0.80	0.53-1.23	
**Tone of content**						.97			.25
	Neutral or disagreement between coders	571	6	1	N/A		1	N/A	
	Positive	528	8	1.01	0.65-1.55		0.84	0.62-1.13	

^a^IRR: incident rate ratio.

^b^*P* values calculated for whole variables using the chi-square test. We controlled for reach by offsetting the negative binomial regression model by the reach of the original posts.

^c^We controlled for reach by offsetting the negative binomial regression model by the reach of the original posts.

^d^N/A: not applicable.

**Table 4:** Reactions to shared posts from each type of original post, before and after controlling for reach of shared posts.

Variable			Reactions, n		Reactions, median		IRR^a^		95% CI		*P* value^b^		IRR (controlling for reach)^c^		95% CI		*P* value^b^
Total			990		0		N/A^d^		N/A		N/A		N/A		N/A		N/A
**Health service**											.003						.02
	1	74		0		1		N/A				1		N/A			
	2	215		0		3.84		1.13-13.02				1.99		0.99-3.98			
	3	701		10		9.19		2.98-28.33				0.87		0.47-1.61			
**First Nations (Australia) content**											.78						.29
	No	231		1		1		N/A				1		N/A			
	Yes—not local	92		0		0.57		0.10-3.25				1.54		0.62-3.84			
	Yes—local	667		0		1.00		0.27-3.62				1.69		0.90-3.18			
**Content source**											.02						.003
	Original content	399		0		1		N/A				1		N/A			
	Other sources	591		10		3.97		1.31-12.05				2.17		1.31-3.61			
**Video**											.21						.57
	No	589		0		1		N/A				1		N/A			
	Yes	401		8		2.17		0.65-7.22				1.18		0.67-2.10			
**Other organizations tagged**											.29						.11
	No	777		0		1		N/A				1		N/A			
	Yes	213		34		2.53		0.45-14.27				1.86		0.87-3.95			
**Hashtagged**											.58						.002
	No	211		1		1		N/A				1		N/A			
	Yes	779		0		0.66		0.16-2.81				0.36		0.19-0.68			
**Tone of content**											.52						.27
	Neutral or disagreement between coders	433		0		1		N/A				1		N/A			
	Positive	557		1		1.40		0.50-3.98				0.75		0.44-1.26			

^a^IRR: incident rate ratio.

^b^*P* values calculated for whole variables using the chi-square test. We controlled for reach by offsetting the negative binomial regression model by the reach of the shared posts.

^c^We controlled for reach by offsetting the negative binomial regression model by the reach of the original posts.

^d^N/A: not applicable.

Shared posts from original posts with content from nonlocal sources (rather than original content created by the health service) attracted more reactions, which persisted after controlling for reach and other post characteristics (IRR 1.57, 95% CI 1.08-2.27; *P*=.02; Multimedia Appendix 3). Shared posts from health services 2 and 3 (compared with health service 1) had more reactions, but the magnitude of this association decreased after adjusting for reach and became nonsignificant once other characteristics were included in the model (Multimedia Appendix 3).

## Discussion

We found that reach and sharing of posts was largely associated with the health service posting the content. This is unsurprising, given that only 1 service had an established Facebook presence before the study started. Otherwise, we found mixed evidence about other factors, including the use of First Nations–specific content, positive emotional content, video, tagging, and use of hashtags.

A counter-intuitive finding was that reactions to both original and shared posts were associated with content from other sources, not original content. A potential explanation for this could be the lower production quality of locally generated content. However, in this study, many of the posts with locally produced content were of high production quality, including being designed to be of appropriate length and tone for use on social media. Much of the locally generated content was developed and produced with the assistance of a former journalist and author VK, who has extensive professional experience in this area. Given that production quality is unlikely to be an issue, the results could be explained by 2 main factors: novelty and relevance.

Content from other sources may have had a higher novelty value than the local content; other research has shown that novelty can be an important factor in generating engagement [49]. This result may also reflect an effort to be particularly selective about choosing content from other sources, particularly when it contained nonlocal content, which is perceived to be specifically relevant to the local community. Our previous research [15,50] and research by others [44] has found that sharing posts with content obtained from other sources but with a personalized message and posted by a close and trusted family member or friend can be perceived as localized, particularly if the content is highly resonant for the local context (for example, a post that conveys a commonly encountered scenario). Together, these factors may explain why content from other sources generated greater engagement. Further research is required to determine which of these factors explains our results and whether content creators can leverage them to boost engagement with their content.

The more mixed results for First Nations–specific content, both local and nonlocal, may be due to less selectivity being applied when choosing which content to post. It is also possible that, despite local branding and language, and high production values, the generic nature of the Australian government posts had a lower novelty value than other posts. It may also have been perceived as government content, which we have previously found is less trusted than content that is genuinely local [15].

Another unexpected finding was that positive content was associated with lower reach than posts that were coded as neutral and/or contested. Australia’s First Nations people have called for change from deficit discourse to strengths-based messages and research approaches [51]. In our previous research, participants selected positive content for sharing, and the importance of contributing to a supportive environment was highlighted [15,34,50]. However, those studies explored decision making for sharing posts on personal pages, which may differ from the perceptions of posts by organizations. An evaluation of an Australian state government health promotion campaign’s Facebook page found that users expressed a preference for positive content [49], but such content was not associated with higher engagement [30]. The lower reach achieved by positive content in our study may reflect the fact that the coding of most of the remaining posts was contested between the 2 coders or was coded as neutral because it included both positive and negative elements. It may be that unambiguously positive posts were perceived as less interesting or noteworthy than the posts with more ambiguous emotional content. Neutral and contested posts may have contained elements more likely to challenge and stimulate interpersonal discussion. This is important, as interpersonal communication stimulated by exposure to mass media antismoking campaigns is a factor in quitting intentions and behavior [52,53]. In previous research, we have also found evidence that this occurs in response to Facebook posts from friends and family about smoking [15]. We would not recommend avoiding posts with a positive tone based on these new data. Given that people tend to state a preference for positive content, it is likely that this is important for generating a positive connection to a page and receptiveness to messages posted. Therefore, positive content is likely to be complementary to a negative tone or mixed posts that may generate greater engagement.

Although our results were inconclusive, we were able to identify some areas for further research. First, as only 2 posts in our study received paid boosts, we did not perform a separate analysis of these factors. Previous research has found that the use of paid posts can significantly boost reach; however, it has also shown that organic (nonpaid) reach is associated with generating greater page engagement per person reached [30], suggesting that high quality, relevant, and appealing content is still essential if a Facebook campaign is to be successful. Other research has found that page administrators can leverage contextual factors that promote user engagement with pages (eg, the perceived trustworthiness of page, user patterns of use), but these factors cannot be controlled by page administrators [49]. Given the unique role of ACCHOs in delivering health services to First Nations Australians [54,55], posts by ACCHOs may generate engagement because they are seen as a worthy initiative.

The strengths of this study include that the content posted was selected and posted by the partner ACCHOs, based on what they perceived as relevant for their communities. Posting was integrated into routine work, ensuring that the approach was pragmatic and sustainable beyond this study. There were also a number of notable limitations. These included the fact that we did not perform separate analyses for different types of reactions, such as likes and comments, due to the small number of posts and that we were unable to extend our analysis beyond metrics of lower-level engagement, according to a spectrum of engagement outcomes as has been performed in other research [26]. Furthermore, we examined only postlevel data, not the impact of posts on page-level data. In addition, the sparsely populated geographical area, sample size (number of posts), and short duration limit the generalizability of our results. Finally, data from end users, which could have provided insights into the types of content that would be most likely to generate engagement, are not included in this paper.

### Conclusions

Overall, we did not find a definitive set of characteristics that were clearly associated with reach and engagement. The exception was that content from other sources was associated with higher engagement than original content, which was an unexpected finding. However, it is important to note that our results do not suggest that community-based, localized content should be avoided. Similarly, although posts perceived by community members to have a positive tone generated less engagement, we do not suggest that these should be avoided. Rather, positive tone posts could be complementary to posts with characteristics that are likely to generate greater interaction. One of the key appeals of social media, specifically Facebook, is its ability to reach relatively small populations and to tailor content accordingly. For Australia’s First Nations people, it can help with building social capital and connection. This study contributes to an understanding of the role of localized, community-based social media efforts and how these can be implemented. However, additional research is needed to understand the extent to which social media content can influence behavior.

## Appendix

Multimedia Appendix 1 Total shares of post controlling for reach, before and after adjusting for all other variables.

Multimedia Appendix 2 Total reactions to original posts controlling for reach, before and after adjusting for all other variables.

Multimedia Appendix 3 Total reactions to shared posts controlling for reach, before and after adjusting for all other variables.

## Abbreviations

ACCHO: Aboriginal Community Controlled Health Service
IRR: incident rate ratio
