# Supplementary material for: Facebook-Based Social Marketing to Reduce Smoking in Australia’s First Nations Communities: An Analysis of Reach, Shares, and Likes
Source: J Med Internet Res. 2020 Dec 10;22(12):e16927. doi: 10.2196/16927 (PMC7759443; doi:10.2196/16927)
Supplement: Multimedia Appendix 2 [file jmir_v22i12e16927_app2.docx]

Multimedia Appendix 2. Table S2: Total reactions to original posts controlling for reach, before and after adjusting for all other variables.

**Table S2. Total reactions to original posts controlling for reach, before and after adjusting for all other variables**

|  | **n** | **IRR, controlling for reach** | **CI** | **Adjusted IRR** | **CI** |
| --- | --- | --- | --- | --- | --- |
| **Health Service** |  | *P=.25* |  | *P=.047* |  |
| 1 | 334 | 1 |  | 1 |  |
| 2 | 162 | 1.3 | (0.88-1.94) | 1.11 | (0.74-1.67) |
| 3 | 603 | 0.95 | (0.67-1.35) | 0.71 | (0.49-1.03) |
| **First Nations (Australia) content** |  | *P=.003* |  | *P=.42* |  |
| No | 182 | 1 |  | 1 |  |
| Yes – not local | 92 | 0.96 | (0.59-1.58) | 0.82 | (0.51-1.31) |
| Yes – local | 825 | 1.71 | (1.19-2.46) | 1.11 | (0.75-1.65) |
| **Content source** |  | *P<.001* |  | *P=.02* |  |
| Original content | 517 | 1 |  | 1 |  |
| Other sources | 582 | 1.89 | (1.40-2.56) | 1.57 | (1.08-2.27) |
| **Video** |  | *P=.002* |  | *P=.11* |  |
| No | 661 | 1 |  | 1 |  |
| Yes | 438 | 1.68 | (1.21-2.34) | 1.35 | (0.94-1.95) |
| **Other orgs tagged** |  | *P=.02* |  | *P=.17* |  |
| No | 890 | 1 |  | 1 |  |
| Yes | 209 | 1.74 | (1.09-2.78) | 1.37 | (0.88-2.13) |
| **Hashtagged** |  | *P=.31* |  | *P=.85* |  |
| No | 158 | 1 |  | 1 |  |
| Yes | 941 | 0.8 | (0.53-1.23) | 1.04 | (0.69-1.57) |
| **Tone of content** |  | *P=.25* |  | *P=.73* |  |
| Neutral or disagreement between coders^a^ | 571 | 1 |  | 1 |  |
| Positive | 528 | 0.84 | (0.62-1.13) | 1.06 | (0.75-1.49) |

P-value calculated for whole variable using chi-square test. We controlled for reach by offsetting the negative binomial regression model by the reach of the original or shared posts.

^a^Includes posts where all coded as neutral content and posts where coders could not agree if positive, neutral or negative. No posts were coded as negative in tone or content by all coders.
